# The Financial Impact of the ‘Zero-Markup Policy for Essential Drugs’ on Patients in County Hospitals in Western Rural China

**DOI:** 10.1371/journal.pone.0121630

**Published:** 2015-03-19

**Authors:** Zhongliang Zhou, Yanfang Su, Benjamin Campbell, Zhiying Zhou, Jianmin Gao, Qiang Yu, Jiuhao Chen, Yishan Pan

**Affiliations:** 1 School of Public Policy and Administration, Xi’an Jiaotong University, Xi’an, China; 2 Department of Global Health and Population, Harvard School of Public Health, Boston, Massachusetts, United States of America; 3 Bryn Mawr College, Bryn Mawr, Pennsylvania, United States of America; 4 School of Public Health, Xi’an Jiaotong University Health Science Center, Xi’an, China; 5 Ankang Municipal Development and Reform Commission, Ankang, China; 6 Ningshan County Hospital, Shaanxi, China; 7 Zhenping County Hospital, Shaanxi, China; Old Dominion University, UNITED STATES

## Abstract

**Objective:**

With a quasi-experimental design, this study aims to assess whether the Zero-markup Policy for Essential Drugs (ZPED) reduces the medical expense for patients at county hospitals, the major healthcare provider in rural China.

**Methods:**

Data from Ningshan county hospital and Zhenping county hospital, China, include 2014 outpatient records and 9239 inpatient records. Quantitative methods are employed to evaluate ZPED. Both hospital-data difference-in-differences and individual-data regressions are applied to analyze the data from inpatient and outpatient departments.

**Results:**

In absolute terms, the total expense per visit reduced by 19.02 CNY (3.12 USD) for outpatient services and 399.6 CNY (65.60 USD) for inpatient services. In relative terms, the expense per visit was reduced by 11% for both outpatient and inpatient services. Due to the reduction of inpatient expense, the estimated reduction of outpatient visits is 2% among the general population and 3.39% among users of outpatient services. The drug expense per visit dropped by 27.20 CNY (4.47 USD) for outpatient services and 278.7 CNY (45.75 USD) for inpatient services. The proportion of drug expense out of total expense per visit dropped by 11.73 percentage points in outpatient visits and by 3.92 percentage points in inpatient visits.

**Conclusion:**

Implementation of ZPED is a benefit for patients in both absolute and relative terms. The absolute monetary reduction of the per-visit inpatient expense is 20 times of that in outpatient care. According to cross-price elasticity, the substitution between inpatient and outpatient due to the change in inpatient price is small. Furthermore, given that the relative reductions are the same for outpatient and inpatient visits, according to relative thinking theory, the incentive to utilize outpatient or inpatient care attributed to ZPED is equivalent, regardless of the 20-times price difference in absolute terms.

## Introduction

Medicines are frequently selected, evaluated, used, and spoken about in low-resource settings in ways that are strongly context-dependent [1]. Medicines are not only symbols of healing and hope but are also valuable and sometimes unaffordable commodities within a market dominated by global pharmaceutical companies [1–3]. People in low-resource settings make triage decisions about which medicines to seek, not necessarily on the basis of ‘best practices,’ but rather on cost, as well as aesthetics and palatability [4]. While aesthetic and palatability concerns are more locally engrained, the issue of cost can be addressed effectively by policy making [5]. For instance, decreasing user fees for services and drugs has been shown to increase uptake of care in some low and middle-income countries [6]. The extent to which medicines become available, accessible and affordable to people is deeply impacted by a country’s policy on essential drugs, which ensures the basic access of drugs at low cost [7]. Over 150 countries, most of which are developing countries, have established a national essential medicines list [8]. While it is believed that lowering drug costs increases access, reviews of pharmaceutical policies in developing countries have found virtually no evidence of this [9]. China, which is undergoing rapid health reform, provides a ripe context to investigate the impact of lowering essential drug cost on access to drugs and health-seeking behavior.

China has undergone transformational health reform since 2009. At the heart of this reform have been the reconstruction of a national primary healthcare system and the enhancement of insurance programs targeting low-income citizens [10]. The Chinese Central Government proposed the development of a national essential medicines system, and made it a top priority in health reform [11]. This effort was bolstered by the National Essential Medicines Policy (NEMP), which aims to increase the availability of cost-effective medicines [12]. These efforts represent a major attempt to “broaden” access to essential drugs in the country and ultimately improve health equity [13]

A recent study examining health equity in rural China shows that utilization of healthcare—both inpatient and outpatient—had been largely pro-rich between 1993 and 2008 [14]. This inequity existed partly because drug costs from both inpatient and outpatient care pose a significant burden to low-income people. There is increasing empirical evidence showing that drug costs are primary contributors to medical impoverishment [15–17]. Patients with chronic conditions frequently visit hospitals and can incur high medication costs that are not covered under the catastrophic coverage model of the New Cooperative Medical Schemes (NCMS). Further, Yip and Hsiao et al. found that 11.6% of the rural poor households became impoverished due to outpatient expenses related to chronic disease [17]. To address the financial burden experienced by patients in rural settings, China hopes to match its great success in “broadening” health coverage with a major “deepening” of health care [13]. A critical part of deepening care is making essential drugs more affordable for patients [18].

In an effort to make essential medicines affordable to more people, the Chinese government passed the Zero-markup Policy for Essential Drugs (ZPED). The goals of the policy are to contain the costs of medicines and reduce the financial burden to the public. Since 2009, ZPED has been implemented in primary healthcare institutions, including township health institutions and villages in most provinces of China. Previous research investigating the effect of ZPED at township and village levels has been very promising. For example, Sun et al. showed that per-visit outpatient and inpatient expenses were reduced in six township health centers in Anhui Province [19]. Research by Wang et al. investigated township health institutions in eastern, central, and western China and echoed the results by Sun et al. [19, 20]. Chen et al. conducted another study in Anhui Province with a larger sample size and found a reduction of cost per prescription in 88 primary health institutions [21]. Further, Yang et al. found a reduction in cost per prescription in Hubei province [22]. Song et al. and Shi et al. reached the same conclusions in Shandong province and Beijing, respectively [23, 24]. Lastly, a 2014 study by Song et al. found that the new policy led to price reductions in essential medicines in 149 primary healthcare centers across four Chinese provinces [25].

With promising results at the township and village levels, it is critical to now look at public hospitals at the county level and higher levels, where ZPED has been in a pilot phase for most of the provinces to date.

County public hospitals, which undertake the task of curing patients with common medical conditions, are the main provider of healthcare in rural China. The fourth National Health Service Survey shows that more than 48% of patients were hospitalized in county hospitals [26]. Moreover, statistics from the Ministry of Health in China show that the medical expense in county hospitals consists of 40% of the total medical expenses among all medical institutions [27]. Therefore, in order to control the rapid growth of medical expenses in rural China, it is strategic to hurdle drug expenses in county hospitals. Many studies [19, 20, 28, 29] suggest that ZPED could eliminate the reliance on drug revenue to compensate for the deficit in medical revenue in primary healthcare institutions.

In deepening health system reform in China, it is critical to implement ZPED in county hospitals. At present, ZPED has been piloted for some county hospitals in several provinces. For example, in September 2011, Fuyang County and another 28 counties in Zhejiang implemented ZPED. In June 2011, Shaanxi province made the policy, “Plan of Centralized Purchase of Medicine in Public Hospitals,” and piloted ZPED in county hospitals. However, evidence from a rigorous program evaluation of ZPED at the county hospital level is lacking. While some studies have attempted to assess ZPED at the county level about the effect on the top five most frequent conditions of inpatient services [30] as well as on county hospital revenue and government subsidy levels [31], very little has been done to assess the policy’s financial impact on general patients. To our knowledge, Shen and Wang are the researchers who have investigated ZPED’s financial impact. Shen (2013) conducted an evaluation in Zhejiang province [32] and Wang et al. did research in Henan province [33]. The results from these two studies are contradictory to each other. Shen (2013) showed reduction in per-visit expense in outpatient and inpatient services. However, Wang et al. showed an increased trend instead [33].

Beyond these inconsistent findings in evaluating ZPED at the county level, there are important limitations in the previous research: 1) nearly all the research focused on the community-level medical institutions and few studies evaluated ZPED in county-level hospitals; 2) most research designs only collected data to conduct pre-post comparisons, which lacks scientific rigor in evaluating the policy; 3) only descriptive statistics methods were used in most of the research and confounding variables like age, gender, marital status and severity of medical condition could not be controlled. In order to overcome these limitations in the previous research and to fill an important research gap, this study aims to assess the impact of ZPED on medical expenses, including outpatient and inpatient expenses, in county hospitals, and to explore how the cost of essential drugs impacts individual behavior and pharmaceutical uptake.

## Methods

### Study design

Ningshan county hospital in Ankang city, Shaanxi province, was selected to be the study object. According to the documents provided by Ningshan county hospital, ZPED was implemented since December 1^st^, 2010 in Ningshan county hospital. Among essential drugs, 44% of them were selected to be subject to the zero-markup policy. From December 1^st^, 2010 to November 11^th^, 2011, in Ningshan county hospital, the cost of the drugs purchased was 7.88 million CNY and the revenue of the drugs sold was 6.96 million, in which the cost and revenue of zero-markup drugs were 1.27 million and 1.1 million, respectively, accounting for 16.12% and 15.8% of the total cost and revenue. In 2011, patients benefited from 194 thousand CNY due to ZPED, according to hospital financial records. On average, the markup rate decreased by 2.46% in 2011.

In this quasi-experimental study, Ningshan county hospital was the experimental group, compared to Zhenping county hospital, the control group. Zhenping county hospital in Shaanxi province was selected as the control group because of the following reasons. First, comparability between Ningshan county and Zhenping county was indicated in administrative district, geography, income per capita, and health resources, etc. (Table 1). Second, comparability between Ningshan county hospital and Zhenping county hospital was indicated in the size of hospital, clinical departments, and medical personnel, etc. (Table 2). Third, ZPED had not yet been implemented in Zhenping county hospital in 2011, making that hospital and year an excellent control for the study.

**Table 1:** The basic information of Ningshan county and Zhenping county in 2011.

	Ningshan county	Zhenping county
Administrative district	Ankang, Shaanxi province	Ankang, Shaanxi province
Location	South foot of Qin mountain	North foot of Daba mountain
Acreage	3678 square kilometers	1503 square kilometers
Population	74 thousand	56 thousand
Income per capita for county/town residents	16,609 CNY	16,560 CNY
Income per capita for villagers	4,498 CNY	4,450 CNY
Number of beds per one thousand for hospitalization	3.35	4.36
Number of healthcare professionals per one thousand	3.62	3.43

**Table 2:** Basic information of Ningshan county hospital and Zhenping county hospital in 2011.

	Ningshan county	Zhenping county
Year of establishment	1950	1950
Hospital certificate	Non-profit secondary generalHospital	Non-profit secondary generalhospital
Floor area	6,492 square meters	15,000 square meters
Clinical departments	Medical department, surgical department, department of gynecology and obstetrics, emergency department, anesthesia department, Chinese medicine department, ophthalmology and otorhinolaryngology, department of stomatology, clinical laboratory, radiology department and functional department	Medical department, surgical department, department of gynecology and obstetrics, pediatrics, Chinese medicine department, ophthalmology, department of stomatology, dermatological department and department of physiotherapy
Number of staff	196	173
Doctors	99	68
Nurses	70	63
Number of beds	151	150

### Ethics

Our research team members have no direct contact with human subjects. All the patients’ information collected from medical records was anonymized and de-identified prior to analysis. The study protocol was reviewed and approved by the Ethics Committee of Xi’an Jiaotong University Health Science Center.

### Data sources

Summary statistics of the hospitals’ basic information include the hospital certificate, department, staff, and number of beds of Ningshan county hospital and Zhenping county hospital. The operation reports include the two hospitals’ outpatient expense per visit, inpatient expense per visit, and drug expense per visit.

In random sampling, 1,007 outpatient records in 2010 and 1,007 outpatient records in 2011 were selected from Ningshan county hospital, which include the outpatients’ demographic information, outpatient expense and its composition. As the information system of outpatient service was not established in Zhenping county hospital, the outpatient records from Zhenping county hospital were not collected in this study. All the inpatient records from 2010 to 2011 were collected in Ningshan and Zhenping county hospitals. In Ningshan county hospital, 2,728 and 3,078 records were collected for years 2010 and 2011, respectively. In Zhenping county hospital, as the information system of inpatient service has not been integrated, the personal information, disease diagnosis, and the inpatient expense were in separate systems. The data from the three systems were merged by admission number. Finally, 1,711 and 1,722 records in 2010 and 2011, respectively, were used in data analysis, which is about 39% and 38% of the total inpatient records in 2010 and 2011, respectively. The medical records include the inpatient’s basic information, disease diagnosis, inpatient expense, and its composition.

### Outcome variables

**Fig 1:**
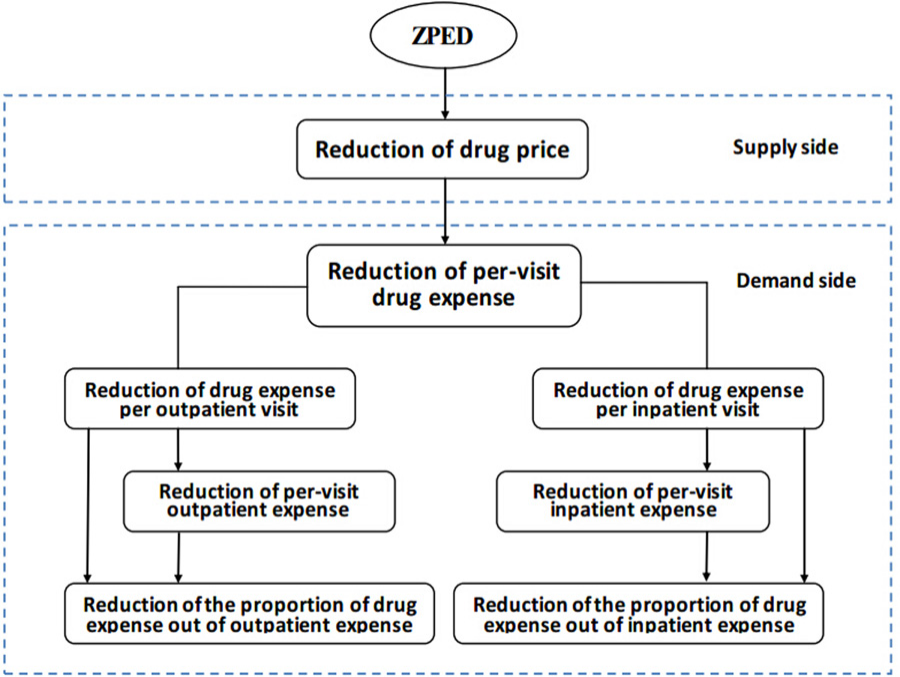
Theoretical framework.

Indicators which were employed to measure the financial impact of ZPED include: per-visit outpatient expense, drug expense per outpatient visit, the proportion of drug expense out of outpatient expense, per-visit inpatient expense, drug expense per inpatient visit, and the proportion of drug expense out of inpatient expense. We hypothesize that the per-visit outpatient and inpatient expense, drug expense and the proportion of drug expense will be reduced after implementing ZPED. A theoretical framework on how ZPED is proposed to influence the outcome indicators is shown in Fig. 1.

### Statistical methods

The method of difference—in—difference (DID) [34] was employed to analyze the effects of ZPED. Because both the hospital-level data and individual-level data are available, we conduct analysis in two approaches, accordingly. We denote the DID using hospital-level data as ‘Hospital-data DID’ and the one using individual-level data as ‘Individual-data DID.’

The implementation effect of ZPED is:

$$\beta =(y_{1,1}-y_{1,0})-(y_{0,1}-y_{0,0})$$ (1)

In Equation (1), for each first subscript, “1” represents treatment-group hospital and “0” represents control-group hospital; for each second subscript, “1” represents the year of 2011 and “0” represents the year of 2010. Accordingly, *y*_1,0_ and *y*_0,0_ are evaluation indicators for treatment group and control group, respectively, in 2010; *y*_1,1_ and *y*_0,1_ are evaluation indicators for the treatment group and control group, respectively, in 2011. The effect of zero-markup policy is captured by*β*.

In order to control both the observable confounding factors, linear regression models were employed to analyze the effects of ZPED [35].

- Dependent variables: inpatient expense, drug expense in inpatient service, the proportion of drug expense in inpatient expense.
- Independent variables: *policy* (*policy* = 1 means treatment-group hospital,*policy* = 0 means control-group hospital), *year* (*year* = 1 means the year of 2011, *year* = 0 means the year of 2010).
- Control variables: age, gender, marital status, occupation, health insurance, severity of illness, surgery, treatment outcome.

The regression model is as follow:

$$y=\beta_{0}+\beta_{1}policy+\beta_{2}year+\beta_{3}policy\cdot year+\sum_{m=1}\delta_{m}x_{m}+\mu$$ (2)

In Equation (2), *y* is the dependent variable, *policy* and *year* are independent variables,*policy Year* is the interaction term, *x*_*m*_ is the control variable, *μ* is a residual term. *β*_3_, the coefficient of the interaction term, is the effect of ZPED. If no control variables are included in the regression model, *β*_3_ is the estimate of DID:

$$\beta_{3}=({\bar{y}}_{1,1}-{\bar{y}}_{1,0})-({\bar{y}}_{0,1}-{\bar{y}}_{0,0})$$ (3)

The notations in this equation are the same as those in Equation (1). When the control variables are included in Equation (2), the estimate of *β*_3_ would become more sophisticated.

## Results

### Testing the assumption of DID

DID is based on the assumption that, without the intervention, the trend of the outcome variables from treatment group and control group should be similar. Of note, Chen et al. did apply the DID model in their study in China; however, the assumptions of DID were left unexamined [36]. In this study, the assumptions are satisfied with the following evidence.

First, Ningshan county and Zhenping county are similar in terms of the administrative district, geography, income per capita, and health resources. Moreover, there is similarity between Ningshan county hospital and Zhenping county hospital in terms of the certificate of hospital, clinical departments, and medical resources.

**Fig 2:**
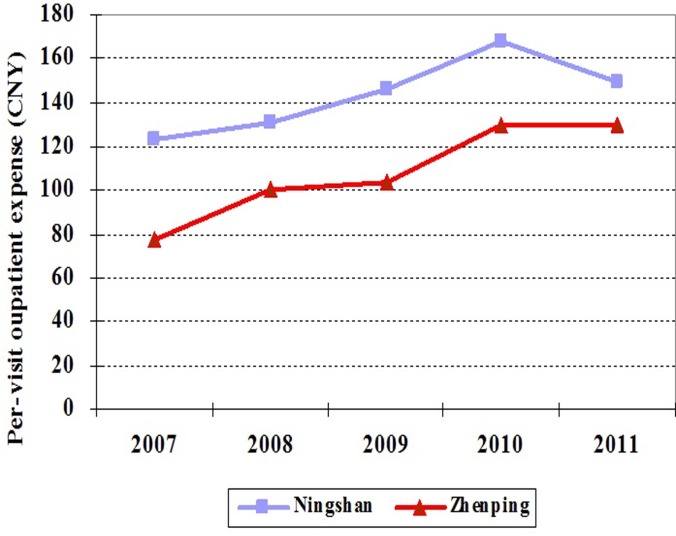
The changing trends of outpatient expense in Ningshan and Zhenping county hospitals from 2007 to 2011.

**Fig 3:**
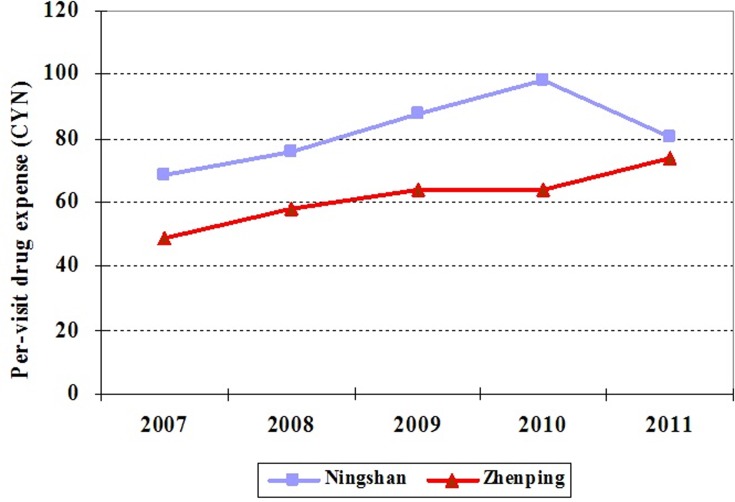
The changing trends of drug expense per outpatient visit in Ningshan and Zhenping county hospitals from 2007 to 2011.

Second, in order to demonstrate that the trends between two hospitals are similar for all outcome variables, we collected data from 2007 to 2011. Fig. 2 and Fig. 3 show that, for both per-visit outpatient expense and drug expense per outpatient visit, from 2007 to 2010, the trends in Ningshan county hospital are similar to those in Zhenping county hospital, which is consistent with the hypothesis of DID.

**Fig 4:**
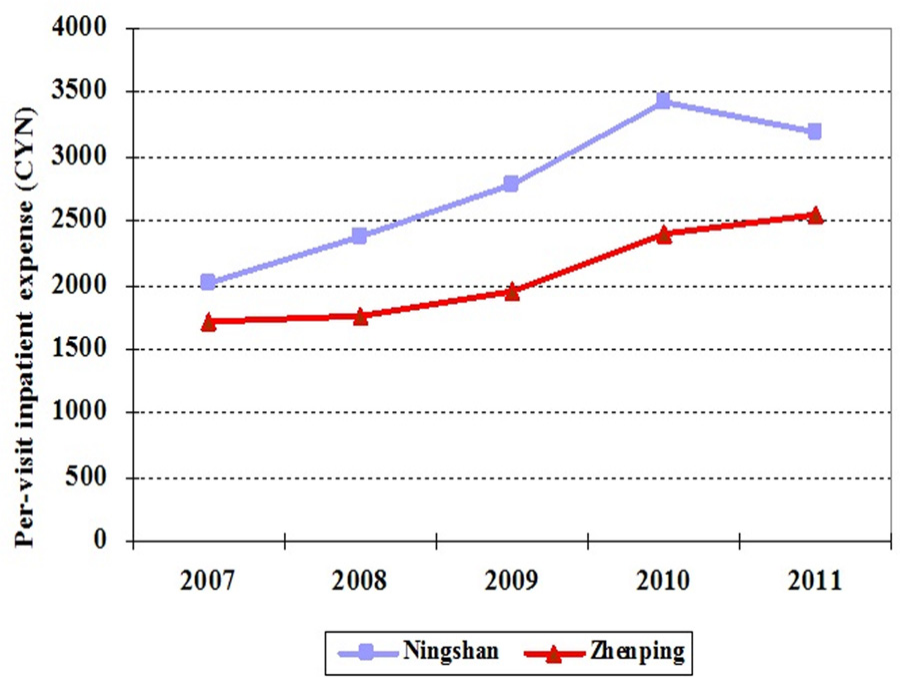
The changing trends of inpatient expense in Ningshan and Zhenping county hospitals from 2007 to 2011.

**Fig 5:**
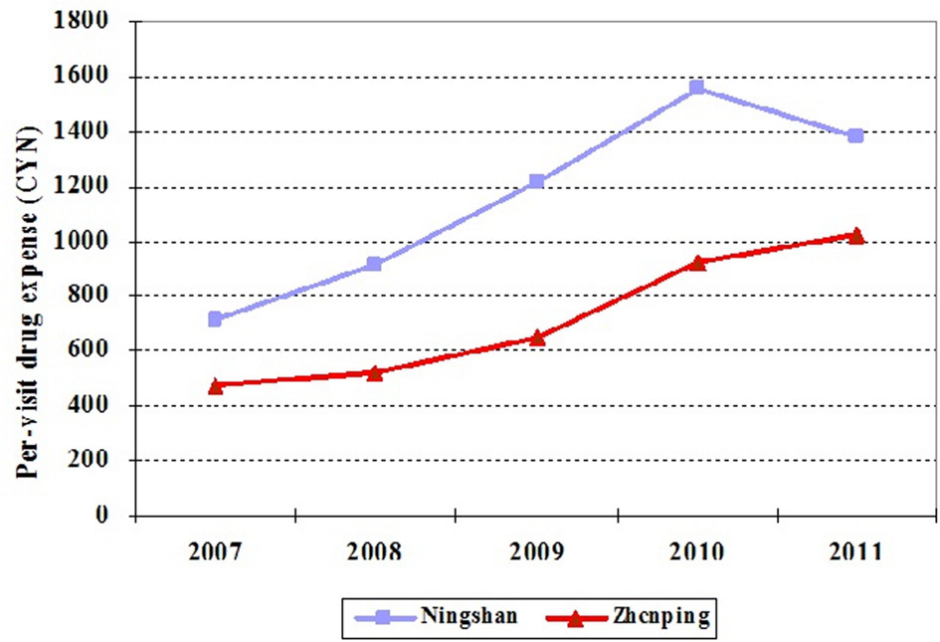
The changing trends of drug expense per inpatient visit in Ningshan and Zhenping county hospitals from 2007 to 2011.

In addition, Fig. 4 and Fig. 5 show that the trends of per-visit inpatient expense and drug expense per inpatient visit are similar in two county hospitals from 2007 to 2010 as well. Therefore, DID can be applied to evaluate the effects of ZPED on per-visit medical expense in the county hospital.

### Results from hospital-data DID

From the results of second-order difference, as shown in Table 3, with the implementation of ZPED in Ningshan county hospital, the per-visit outpatient expense, the drug expense per outpatient visit, and the proportion of drug expense in outpatient expense dropped by 19.02 CNY (3.12 USD), 27.20 CNY (4.46 USD) and 11.73 percentage points, respectively. The per-visit inpatient expense, the drug expense per inpatient visit, and the proportion of drug expense in inpatient expense declined by 389.11 CNY (63.87 USD), 278.42 CNY (45.70 USD) and 3.9 percentage points, respectively.

**Table 3:** The effects on per-visit outpatient and inpatient expense.

	Per-visitexpense (CNY)		Per-visit drugexpense (CNY)		Proportion (%)	
	Ningshan	Zhenping	Ningshan	Zhenping	Ningshan	Zhenping
Outpatient expense						
2010	168.00	130.25	98.05	63.99	58.36	49.13
2011	148.96	130.23	80.44	73.58	54.00	56.50
D1	-19.04	-0.02	-17.61	9.59	-4.36	7.37
DID	-19.02		-27.20		-11.73	
Inpatient expense						
2010	3418.78	2394.03	1549.81	919.98	45.33	38.43
2011	3182.81	2547.18	1376.59	1025.18	43.25	40.25
D1	-235.97	153.14	-173.22	105.20	-2.08	1.82
DID	-389.11		-278.42		-3.90	

### Results from individual-data DID

As the information of individual outpatient records from Zhenping county hospital was not collected, only the data of individual outpatient records from Ningshan county hospital in the years of 2010 and 2011 is utilized to conduct regressions. Three regressions were employed to analyze the effects of ZPED, in which the dependent variables are per-visit outpatient expense, drug expense per outpatient visit, and the proportion of drug expense in outpatient expense, the independent variable is year, and the control variables are gender, age, surgery, etc. The descriptions of the dependent variables are shown in Table 4. From the year of 2010 to 2011, the per-visit outpatient expense, per-visit drug expense, and the proportion of drug expense in outpatient expense declined by 16.53 CNY (2.71 USD), 17.37 CNY (2.85 USD), and 5.01 percentage points, respectively. The descriptions of independent variables are shown in Table 5.

**Table 4:** Outpatient services: description of dependent variable in the linear regression models.

	Sample	Per-visit outpatientexpense (CNY)	Per-visit drugexpense (CNY)	Proportion (%)
2010	1007	166.79	99.10	59.41
2011	1007	150.26	81.73	54.40
Total	2014	158.53	90.42	57.04

**Table 5:** Outpatient services: the description of independent variables in the linear regression models.

Variables	Description	Percentage (%)
Year		
2010*	2010 = 1, 2011 = 0	50.0
2011	2011 = 1, 2010 = 0	50.0
Gender		
Female*	Female = 1, male = 0	40.4
Male	Male = 1, female = 0	59.6
Age		
0 to 6 years old*	0 to 6 years old = 1, other = 0	36.8
7 to 17 years old	7 to 17 years old = 1, other = 0	20.2
18 to 40 years old	18 to 40 years old = 1, other = 0	30.0
41 to 65 years old	41 to 65 years old = 1, other = 0	11.6
More than 65 years old	More than 65 years old = 1, other = 0	1.5
Surgery		
No surgery*	No surgery = 1, surgery = 0	98.2
Surgery	Surgery = 1, no-surgery = 0	1.8

Note: * stands for the reference group in regressions.

The results of regressions are shown in Table 6. After controlling for the variables of gender, age and surgery, the per-visit outpatient expense, drug expense per outpatient visit, and the proportion of drug expense in outpatient expense declined in a statistically significant manner, by 15.32 CNY (2.51 USD), 17.14 CNY (2.81 USD) and 7.35 percentage points, respectively.

**Table 6:** Outpatient services: results of the linear regression models.

	Per-visit outpatient expense		Per-visit drug expense		Proportion	
	Coefficients	S. E.	Coefficients	S. E.	Coefficients	S. E.
2011	-15.32**	6.29	-17.14***	5.36	-7.35***	1.62
Male	15.16**	6.41	0.22	5.47	-2.45	1.65
7 to 17 years old	-0.28	8.63	-4.44	7.36	-2.66	2.22
18 to 40 years old	-0.80	7.75	-3.99	6.61	-2.52	2.00
41 to 65 years old	44.68***	10.49	43.49***	8.95	11.80***	2.70
More than 65 years old	16.51	25.98	34.58	22.16	14.04**	6.69
Surgery	148.24***	23.51	15.42	20.06	-23.63***	6.06
F	10.41		6.33		10.42	
p	<0.001		<0.001		<0.001	

Note: *significant at 10%,

**significant at 5%,

***significant at 1%.

Using the data of inpatient records from Ningshan and Zhenping county hospitals in years 2010 and 2011, DID with control variables in regressions was employed to analyze the effects of ZPED. From Table 7, in Ningshan County hospital, compared with 2010, the per-visit inpatient expense, drug expense per inpatient visit, and proportion of drug expense in inpatient expense declined in 2011. In Zhenping county hospital, the per-visit inpatient expense and the drug expense per inpatient visit increased from 2010 to 2011. The descriptions of independent variables are shown in Table 8.

**Table 7:** Inpatient services: the description of dependent variables in the linear regression models.

	Sample	Per-visit inpatientexpense (CNY)	Per-visit drugexpense (CNY)	Proportion (%)
Ningshan county hospital				
2010	2728	3482.98	1565.12	41.71
2011	3078	3203.47	1368.07	37.58
Total	5806	3334.80	1460.66	39.52
Zhenping county hospital				
2010	1711	2738.23	1088.14	35.70
2011	1722	3028.73	1216.78	35.25
Total	3433	2883.94	1152.67	35.47

**Table 8:** Inpatient services: description of independent variables in the linear regression models.

Variables	Description	Percentage (%)
Year		
2010*	The year of 2010 = 1, the year of 2011 = 0	48.1
2011	The year of 2011 = 1, the year of 2010 = 0	52.0
County hospital		
Zhenping	Zhenping = 1, Ningshan = 0	37.2
Ningshan	Ningshan = 1, Zhenping = 0	62.8
2011*Ningshan	The interaction of 2011 and Ningshan = 1, other = 0	33.3
Gender		
Female*	Female = 1, male = 0	51.5
Male	Male = 1, female = 0	48.5
Age	The age of inpatient	40.1
Occupation		
Peasant*	Peasant = 1, other = 0	62.3
Worker	Worker = 1, other = 0	2.8
Government officer	Government officer = 1, other = 0	4.5
Teacher	Teacher = 1, other = 0	11.9
Student	Student = 1, other = 0	2.7
Unemployed	Unemployed = 1, other = 0	4.9
Child	Child = 1, other = 0	4.8
Other occupation	Other occupation = 1, other = 0	6.1
Marital status		
Married*	Married = 1, other = 0	74.0
Unmarried	Unmarried = 1, other = 0	23.5
Divorced	Divorced = 1, other = 0	0.2
Widowed	Windowed = 1, other = 0	2.3
Other marital status	Other marital status = 1, other = 0	0.0
Health insurance		
UEMI*	UEMI = 1, other = 1	8.9
URMI	URMI = 1, other = 1	2.6
NCMS	NCMS = 1, other = 0	64.9
Insurance for gov officers	Insurance for gov officers = 1, other = 0	0.9
Other health insurance	Other health insurance = 1, other = 0	1.4
Out-of-pocket	Out-of-pocket = 1, other = 0	21.5
Severity of medical condition		
Not-at-all severe*	Not-at-all severe = 1, other = 0	72.3
Severe	Severe = 1, other = 0	22.5
Very serious	Very serious = 1, other = 0	5.3
Outcome of hospitalization		
Healed*	Healed = 1, other = 0	50.2
Improved	Improved = 1, other = 0	28.5
No change	Not healed = 1, other = 0	2.0
Death	Death = 1, other = 0	0.6
Other outcome	Other outcome = 1, other = 0	18.8
Surgery or not		
No surgery*	No surgery = 1, other = 0	73.3
Surgery	Surgery = 1, other = 0	26.7

Table 9 shows that the coefficients of the interaction of 2011 and Ningshan in the models with per-visit inpatient expense, drug expense per inpatient visit, and proportion of drug expense in inpatient expense as dependent variables are statistically significant. After controlling for the confounding factors, the per-visit inpatient expense, drug expense per inpatient visit, and the proportion of drug expense out of inpatient expense dropped by 399.6 CNY (65.59 USD), 278.7 CNY (45.75 USD) and 3.92 percentage points, respectively.

**Table 9:** Inpatient services: the results of linear regression.

	Per-visit inpatient expense		Per-visit drug expense		Proportion	
	Coefficients	S.E	Coefficients	S.E	Coefficients	S.E
2011	122.5	110.4	71.3	61.4	0.17	0.52
Ningshan	-607.6***	121.4	-50.2	67.5	7.66***	0.58
2011*Ningshan	-399.6***	139.9	-278.7***	77.8	-3.92***	0.66
Male	499.0***	72.9	256.7***	40.6	3.68***	0.35
Age	52.8***	2.4	30.2***	1.3	0.29***	0.01
Worker	811.5***	228.7	316.3**	127.2	1.32	1.09
Government officer	499.6***	185.6	351.0***	103.2	3.21***	0.88
Teacher	742.0***	158.7	580.7***	88.3	8.07***	0.75
Student	491.2**	242.9	288.1**	135.1	2.64**	1.15
Unemployed	292.9*	168.7	199.4**	93.9	3.80***	0.80
Child	392.4*	215.0	340.4***	119.6	12.57***	1.02
Other occupation	1583.3***	171.5	726.7***	95.4	1.71**	0.81
Unmarried	69.9	139.0	59.3	77.3	5.25***	0.66
Divorced	75.4	730.9	-76.1	406.6	1.06	3.47
Widowed	-419.2*	228.6	-208.1	127.2	-0.31	1.09
Other marital status	1627.0	3177.8	812.6	1767.7	-7.37	15.08
URHI	-715.7***	258.1	-438.1***	143.6	1.29	1.23
NCMS	-822.5***	164.9	-672.2***	91.8	-0.44	0.78
Insurance for gov officers	4967.5***	379.7	1512.5***	211.2	-13.47***	1.80
Other health insurance	-550.6*	330.3	-648.5***	183.7	1.07	1.57
Out-of-pocket	-367.3**	166.9	-452.2***	92.9	-0.10	0.79
Severe condition	0.7	81.6	6.5	45.4	-0.20	0.39
Very severe condition	1563.4	156.7	488.7***	87.2	-6.06***	0.74
Improved	-357.3	84.8	-57.3	47.2	1.71***	0.40
Not healed	-503.9	244.8	-379.8***	136.2	-11.00***	1.16
Death	340.3	434.0	55.3	241.4	-13.51***	2.06
Other outcome	-1340.2	103.8	-654.2***	57.7	-16.08***	0.49
Surgery	2509.1	85.8	445.0***	47.7	-15.46***	0.41
F	122.28		101.00		335.83	
p	<0.001		<0.001		<0.001	

Note: *significant at 10%,

**significant at 5%,

***significant at 1%.

Table 10 shows the reduction of expense in relative terms. For outpatient visits, the expense reduced by 11% according to hospital-data DID and by 9% according to pre-post comparison in regression. For inpatient visits, the expense reduced by 11% from both hospital-data and individual-data DID. Assuming the results for outpatient expense remains the same from hospital-data and individual-data DID, the estimate of relative change in outpatient expense from hypothetical individual-data DID is 11%.

**Table 10:** Reduction of expense in relative terms.

	Baseline expense in 2010(CNY)	Absolute change(CNY)	Relative change
Hospital-level DID			
Outpatient expense	168	-19.02	-11%
Inpatient expense	3418.78	-389.11	-11%
Regressions			
Outpatient expense	166.79	-15.32	-9%
Inpatient expense	3482.98	-399.6	-11%

In sum, in absolute terms, the total expense per visit reduced by 19.02 CNY (3.12 USD) for outpatient services and 399.6 CNY (65.60 USD) for inpatient services. In relative terms, the reduction of expense per visit is 11% for both outpatient and inpatient services. The drug expense per visit dropped by 27.20 CNY (4.47 USD) for outpatient services and 278.7 CNY (45.75 USD) for inpatient services. The proportion of drug expense out of total expense per visit dropped by 11.73 percentage points in outpatient visits and by 3.92 percentage points in inpatient visits.

## Discussion

### Inpatient expense

Methodologically, the DID method could be only used to control the time-related unobservable confounding variables but could do nothing for the observable confounding variables like gender, age, severity of illness, treatment effect, etc. In order to control both of the observable confounding variables and time-related unobservable confounding variables, DID with control variables in regressions is employed to further analyze the effects of ZPED on inpatient expense. The results are similar with or without controlling for observable variables (see Table 11). It suggests that unobservable confounders rather than demographic characteristics contribute more to the point estimates of outcomes impacted by ZPED.

**Table 11:** The effects of ZPED on inpatient expense by using different methods.

	Per-visit inpatient expense (CNY)	Per-visit drug expense (CNY)	Proportion (%)
Hospital-level DID	-389.11	-278.42	-3.90
Individual-data DID	-399.6	-278.7	-3.92

### Outpatient expense

To analyze the outpatient data, both hospital-data DID and regressions are employed. Without access to the outpatient records in Zhenping county hospital, only data from Ningshan hospital are utilized in regressions for pre-post comparison. The observable confounding variables such as gender, age, and surgery are controlled in regressions. Table 6 shows that control variables such as age and surgery have an impact on the estimates. However, the unobservable confounders remain unaddressed. In the DID, the unobservable and time-related confounding factors are controlled. The results from the DID are different from those from regressions because unobservable confounders are controlled. It is assumed that unobservable confounders rather than demographic characteristics contribute more to the point estimates of ZPED on outpatient expense. From Table 3, the per-visit outpatient expense and the per-visit drug expense drop by 19.02 CNY (3.12 USD) and 27.20 CNY (4.46 USD), respectively. Importantly, this implies that the non-drug expense increased by 8.18 CNY (1.34 USD).

### Patient decision-making

Because ZPED reduces healthcare prices for patients, it has the potential to change the structure of inpatient and outpatient utilization. First, the probability of an inpatient visit is independent from the change of outpatient price [37]. This means that patients’ decision making on whether to utilize inpatient services are determined by factors other than outpatient price. Second, the impact of inpatient price on the probability of outpatient visit is significant and positive. The point estimate was 0.171 from pooled data from nationally representative surveys in 2003 and 2008 [37]. This means that the outpatient visits by the general population will decrease by 2% if the inpatient price is reduced by 11%. A very interesting point made in the Zhou et al. (2011) study is that users of outpatient services are more sensitive than the general population to the change in inpatient price. For users of outpatient services, outpatient visits will decrease by 3.39% if the inpatient price is reduced by 11%. Third, it is necessary to emphasize that in the study conducted by Zhou et al. [37], among nine estimated cross-price elasticity values, only two are significant at the 95% confidence level. In most of the cases, outpatient services and inpatient services are un-exchangeable. Even though in the naïve case that outpatient services are substitutes for inpatient services, the magnitudes of 2% and 3.39% are small. However, these estimates in traditional economics may not hold from the perspective of behavioral economics. Substantial evidence from behavioral economics research has shown that people oftentimes make decisions not based on absolute, but on relative changes in price [38]. This phenomenon is at the heart of relative thinking theory, which states that people are influenced more by relative changes than by absolute changes in a given base line.

## Limitations

There are some important limitations in this study. First, the individual outpatient records were not accessible in Zhenping County, which limits the analysis for outpatient services. Second, due to the segmented health record system for inpatient service in Zhenping county hospital, a part of the inpatient records was lost in the merge of the three datasets, which may introduce selection bias. However, it is reasonable to assume that the lost data are random rather than systematic and selection bias is kept at a minimum, if any. Third, the analysis of drug expense was on all drugs rather than on zero-markup drugs because it is impossible to distinguish zero-markup drugs in patient datasets.

## Conclusion

Quantitative analyses indicate that implementation of ZPED is a benefit for patients in both absolute and relative terms. The reduced per-visit expense holds for both outpatient and inpatient services. This could have implications for the demand and utilization of these services in the future. Implementing ZPED will reduce the per-visit medical expense for patients in both absolute and relative terms. The absolute monetary reduction of per-visit inpatient expense is 20 times of that in outpatient care. According to cross-price elasticity, the substitution between inpatient and outpatient is small due to changes in expense. Furthermore, given that the relative reductions are the same for outpatient and inpatient visits, according to relative thinking theory, the incentive to utilize outpatient or inpatient care attributed to ZPED is equivalent, regardless of the 20-times price difference in absolute terms. As a result, it is more likely that comparative utilization of these two services will not change significantly following implementation of ZPED in county hospitals.
